# 

**Published:** 1871-07

**Authors:** 


ORIGINAL CONTRIBUTIONS.

A Short History of Six Cases of Placenta Prævia. By E. R. Willard, M.D., of Wilmington, Ill. ........ 389
Traumatic Paralysis of the Facial Nerve. Recovery. By Alfred E. Walker, M.D. 397
Report on the Medical Uses of Carbolic Acid. By N. S. Davis, M.D., Chicago, Ill., Chairman of the Committee ....... 399

FOREIGN CORRESPONDENCE.

Letter from Ehrenbreitstein, Germany. By G. W. Goodner ........ 408

CORRESPONDENCE.

Sul. Morphia as a Parturient. By L. D. Robinson. M.D ........ 410
Metro-Peritonitis Again. By Theodore Griffin, M.D. ........ 411

SELECTIONS.

War Typhus and Dysentery. By Rudolf Virchow ........ 432

CLINICAL REPORTS.

Clinical Cases in Surgical Wards of Mercy Hospital. Service of Prof. Andrews. From Notes by M. ........ 430

PROCEEDINGS OF SOCIETIES.

Illinois State Medical Society ........ 413
Quincy Medical Society ........ 426
Military Tcact Medical Association ........ 428

EDITORIAL.

Graded Classes and Consecutive Study in the Medical Colleges ........ 445
Imposition ........ 447
Wisconsin State Medical Soicety ........ 448
A Note of Progress ........ 448
Clinical Surgery in the Royal Infirmary Glasgow, Scotland ........ 449
Acute Synovitis—Incision into the Joint 449
Mortality Report for May, 1871 ........ 450
Report by John H. Rauch, Sanitary Superintendent ........ 451

